# Presence of temporomandibular joint discomfort related to pacifier use

**DOI:** 10.1016/S1808-8694(15)31337-9

**Published:** 2015-10-20

**Authors:** Juliana de Paiva Tosato, Daniela Aparecida Biasotto-Gonzalez, Tabajara de Oliveira Gonzalez

**Affiliations:** 1 Physical therapist, Intern, School of Dental Sciences, Piracicaba - FOP/UNICAMP; 2 Ph.D. in Biology and Bucco-Dental Pathology, FOP/UNICAMP, Professor, Course of Physical Therapy, Centro Universitário Nove de Julho; 3 Master in Rehabilitation, UNIFESP, Professor, Course of Physical Therapy, Universidade de Mogi das Cruzes; Universidade de Mogi das Cruzes - Physical Therapy - Mogi das Cruzes, SP

**Keywords:** temporomandibular dysfunction, pacifier suction, children

## Abstract

**Aim**: The goal of the present study was to analyze if the duration of pacifier use influenced the stomatognathic system in children that did not present any other parafunctional habits. **Study design**: Transversal cohort study. **Material and Method**: To collect data, a questionnaire was used and answered by the mothers of 90 children aged three to seven years old. **Results**: The children were divided into three groups: did not use pacifier; used pacifier until 2 years old; and used pacifier for more than 2 years. Greater prevalence of pain or discomfort in the stomatognathic system was observed among the children who had not used pacifier and the children who had used it for more than 2 years. The prevalence was smaller among the children who used pacifier until 2 years of age. **Conclusion**: Thus, it is concluded that pacifier is important to induce children to perform suction movements, preparing them to the introduction of solid foods. However, if used for a prolonged period of time, it may damage the joint and consequently the child's quality of life.

## INTRODUCTION

Temporomandibular joint (TMJ) is a mobile joint between the temporal bone and the mandible^1^. It is a synovial joint that is anatomically and kinesiologically interrelated with other close joints and the back^2^.

Joint dysfunctions are resultant from abnormal functioning and may surge owing to different reasons, such as posture affections, disharmonic condyle position in relation to the disk, parafunctions, psychological factors, proprioceptive affections, resulting from occlusal imbalances, among others^3^.

Temporomandibular joint dysfunctions (TMJD) are described by Siqueira & Ching^4^ as a group of painful orofacial conditions with functional affections of mastication system. These dysfunctions may lead to different signs and symptoms and in many cases there are muscle pain in the masseter and temporalis, joint pain and earache, among others5., 6.

These TMJD are more frequent in female patients; Correia^7^ and Ramos^8^ and Souza^9^ showed that 97.9% of the affections occur in women and they are followed by characteristic signs and symptoms.

Problems in this joint are normally found in adult life, but they may start early in childhood and be related with the child's habits. Suction, considered a nourishing habit up to the age of 3 years and a bad habit after this age, has been quite frequently studied because it is a common habit and causes significant damage10., 11..

Alamoudi et al.^12^ showed that functional disorders of mastication system are common in children and adolescents and tend to increase in adult life. Thilander et al.^13^ stated that temporomandibular dysfunctions in children also present a multifactorial etiology, creating parafunctional habits and occlusal affections that influence the natural function of mastication muscles.

Thus, parafunctional activities such as using a pacifier, normally more common in girls^14^, may be a trigger of TMJD because they cause anterior open bite, mandible retrusion, maxilla protrusion, excessive overbite, buccal direction of upper incisors, posterior crossbite, high palate and angle deformities^15^.

Martins et al.^16^ carried out a study with children aged 2 to 6 years and concluded that the habit of pacifier suction might trigger dental occlusion anomalies.

Cavassani et al.^17^ found joint disorders in 55.56% of the studied sample, which was formed by children aged 5 to 9 years, which presented suction oral bad habits.

Thus, we can see that oral habits, which are frequent in children, cause imbalance of stomatognathic system and it may surge as an etiological factor of TMJD. Therefore, the present study intended to analyze the time of pacifier use and how it influenced the stomatognathic system of children that did not have parafunctional habits.

## MATERIAL AND METHOD

Before data collection, the project was submitted to the Ethics Committee, Universidade de Mogi das Cruzes. After its approval, we asked for authorization by two schools (one private and one public, both located in the city of São Paulo). We informed the mothers of 150 children about the objectives of the study and they signed the free informed consent term.

We included in the study all children whose mothers answered the questionnaire and that did not present other parafunctional habits in addition to pacifier suction, according to the questionnaire. We excluded children whose mothers did not completely answer the questionnaire or did not want to, and those that presented other parafunctional habits in addition to pacifier use, such as thumb suction, bruxism, nail biting or gum chewing.

Questionnaires were given to the mothers, together with a folder addressing temporomandibular joint disorders and a contact telephone in case of questions.

After analyzing the results, 60 children were excluded because they had other parafunctional habits. We included 90 children aged 3 to 7 years and there were 49 girls and 41 boys.

The questionnaire started with personal data and comprised the following questions:

1. Whether the child had TMJ pain;
2. Whether the child had headache;
3. Whether the child had earache;
4. Whether the child got tired when chewing food;
5. Whether the child presented difficulties to chew food;
6. Whether the child had used a pacifier and up to which age;
7. Whether the child had sucked his thumb;
8. Whether the child locked or made noise with the teeth;
9. Whether the child bit his nails;
10. Whether the child chewed gum.

To analyze the results, children were divided into three groups, according to the time they had used a pacifier:

- Children that had not used a pacifier (33 children);
- Children that had used a pacifier up to the age of 2 (14 children);
- Children that had used a pacifier after the age of 2 years (43 children).

### Analysis of results

After the assessment, we calculated the grade of correlation between the collected variables and duration of pacifier use. To measure and assess the level of correlation between random variables we used Pearson Correlation Coefficient^18^. To calculate this coefficient, we used the following formula:

∑
X
Y
-
(
∑
X
)
(
∑
Y
)
n
[
∑
X
2
-
(
∑
X
)
2
n
]
[
∑
Y
2
-
(
∑
Y
)
2
n
]

Variation field of Coefficient “r” between -1 and +1, being that the interpretation depended on the numeric and signal value.

### Alpha value

We used alpha value (α) equal to 0.05 in the statistical test to reject the null hypothesis.

The objective was to determine the duration of pacifier use and whether it modified the prevalence of signs or symptoms of TMJD. These data are shown in Table 1.

**Table 1:** Correlation between time of pacifier use and mean of number of symptoms of TMJD

Time of use	Mean of symptoms	Pearson
0 month	0.78	———————
24 months	0.35	-1
84 months	0.86	1

## RESULTS

**Graph 1:**
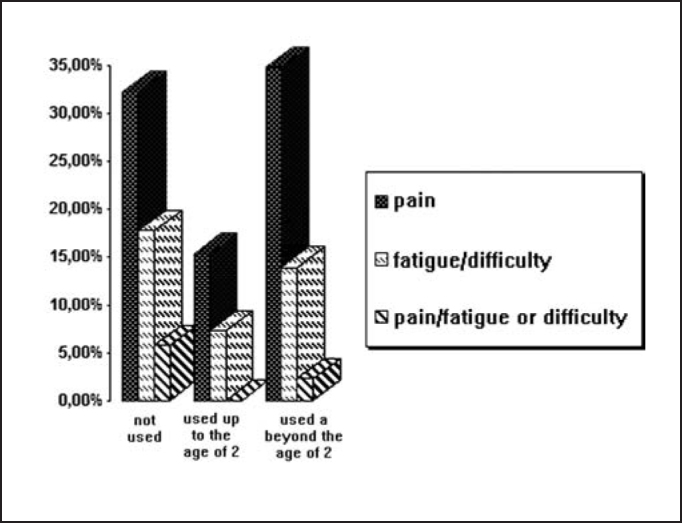
Presence of symptoms of TMJD related with duration of pacifier use.

Among children that had not used a pacifier, 32.2% presented some pain; 17.7% presented fatigue or difficulty to chew food, and 5.8% presented pain, fatigue or difficulty to chew. Out of the children that had used a pacifier up to the age of 2, 15.3% had pain and 7.6 had fatigue or difficulty to chew. Among those that had used a pacifier beyond the age of 2, 34.8% presented pain, 13.9% presented fatigue or difficulty to chew and 2.3% had pain, fatigue and difficulty to chew, as shown in Graph 1.

Upon applying Pearson Correlation Coefficient it was observed that among the children that had not used pacifier and those that had used up to 24 months of age, the increase in time of pacifier use significantly reduced the mean of number of symptoms of TMJD (r = -1.00). Beyond 2 years (considered up to 84 months, which was the maximum duration found for the assessed volunteers), the increase in pacifier use significantly increased the mean in number of TMJD symptoms (r = 1.00) (Table 1).

We also detected higher prevalence of symptoms of dysfunction among girls than boys. Out of the interviewed girls, 53% presented pain or discomfort in the TMJ region, whereas among boys, only 39% presented some symptom of dysfunction.

## DISCUSSION

After the analysis of results obtained through questionnaires answered by the mothers of children, as used in other studies, which demonstrated the validity of responses given by the mothers'^19^, we detected high prevalence of pain or discomfort of stomatognathic system, showing that TMJD is a common disorder among children.

We noticed higher prevalence of TMJD symptoms in girls, which is in agreement with the literature that shows higher incidence in female subjects7., 8., 9..

It was also possible to observe significant correlation between duration of pacifier use and onset of symptoms in children that did not present other parafunctional habits. Pain or fatigue/difficulty to chew was detected mainly in those that had not used a pacifier or used it for up to 2 years. Thus, lack of pacifier use may mean that the child is not prepared for mastication, causing fatigue when chewing some foods. Felício^20^ showed that suction is a necessary muscle exercise that prepares the tongue, lips and mandible for mastication. Thus, if the muscles are not prepared, when solid foods are introduced in the child's diet, he may present muscle fatigue, which is responsible for the symptom of pain.

Excessive use of pacifier, on the other hand, may cause occlusion affections^21^, myofunctional or tongue thrust, interfering in the biomechanics of TMJ. It also causes pain and pain may be the triggering factor of fatigue and difficulty to chew.

In view of the results, we could notice that the pacifier may be important for the child to make suction movements, preparing him for the introduction of solid foods^22^. However, if excessively used, it may impair articulation and, consequently, the quality of life of the child.

## CONCLUSION

It was possible to conclude that the use of pacifier significantly influenced the prevalence of symptoms of TMJD.

## References

[1] Savalle WPM., Steenks MH, Wijer A. (1996). Disfunções da Articulação Temporomandibular do Ponto de Vista da Fisioterapia e da Odontologia..

[2] Okeson JP. (1992). Fundamentos de Oclusão e Desordens Temporomandibulares..

[3] Teixeira ACB, Marcucci G, Luz JGC. (1999). Prevalência das maloclusões e dos índices anamnésicos e clínicos em pacientes com disfunção da articulação temporomandibular.. Rev Odonto USP.

[4] Siqueira JTT, Ching LH. (1999). Bases para Diagnóstico Clínico..

[5] Barbosa GAS (2003). Distúrbios Oclusais: Associação com a Etiologia ou uma Conseqüência das Disfunções Temporomandibulares. JBA.

[6] Magnusson T (1994). Changes in clinical signs of craniomandibular disorders from the age of 15 to 25 years.. J Orofac Pain.

[7] Correia FAS. (1983). Prevalência da sintomatologia nas disfunções da articulação temporomandibular e suas relações com idade sexo e perdas dentais (dissertação)..

[8] Ramos HAD (1992). Sinais e sintomas das disfunções dolorosas da articulação temporomandibular.. Odonto Cad Doc.

[9] Souza JAS. (1980). Síndrome da articulação temporomandibular.. RGO.

[10] Bayardo RE, Mejla JJ, Orozco SLE., Montoya K.B.S. (1996). Etiology of oral habits.. J Dent Child.

[11] Turgeons B, Lachapelle D. (1996). Nutritive and nonnutritive sucking habits.. J Dent Child.

[12] Alamoundi N, Farsi N, Salako N, Feteih R. (1998). Temporomandibular disorders among school children.. J Clin Pediatr Dent.

[13] Thilander B, Rubio G, Pena L, Mayorga C. (2002). Prevalence of Temporomandibular Dysfunction and its association with malocclusion in children and adolescents: an epidemiologic study related to specified stages of dental development.. Angle Orthodontist.

[14] Jersil AJ. (1973). Psicologia da criança..

[15] McDonald HE, Avrry DH. (1986). Odontopediatria..

[16] Martins JCR, Sinimbu CMB, Dinelli TCL, Martins LPM, Rauelli DB. (1998). Prevalência de má oclusão em pré-escolares de Araraquara: relação da dentição decídua com hábitos e nível sócio-econômico.. Revista Dent Press Ortod Ortop Facial.

[17] Cavassani VGS, Ribeiro SG, Nemr NK, Greco AM, Kohle J, Lehn C. (2003). Hábitos orais de sucção: estudo piloto em população de baixa renda.. Revista Brasileira de Otorrinolaringologia.

[18] Toledo GL, Ovalle I. (1995). II Estatística Básica..

[19] Cirano GR (2000). Disfunção de ATM em crianças de 4 a 7 anos: prevalência de sintomas e correlação destes com fatores predisponentes.. RPG.

[20] Felício CM. (1994). Fonoaudiologia na desordens temporomandibulares - Uma ação educativa terapêutica..

[21] Tomita NE, Sheiham A, Bijella VT, Franco LJ. (2000). Relação entre determinantes sócio econômicos e hábitos bucais de risco para más-oclusões em pré-escolares.. Pesq Odont Brás.

[22] André M., Altmann EBC. (1990). Fissuras labiopalatais..

